# Increasing illness severity is associated with global myocardial dysfunction in the first 24 hours of sepsis admission

**DOI:** 10.1186/s13089-022-00282-6

**Published:** 2022-07-28

**Authors:** Robert R. Ehrman, Bryce X. Bredell, Nicholas E. Harrison, Mark J. Favot, Brian D. Haber, Robert D. Welch, Philip D. Levy, Robert L. Sherwin

**Affiliations:** 1 Department of Emergency Medicine, Wayne State University School of Medicine, Detroit, MI USA; 2 Department of Emergency Medicine, Indiana University School of Medicine, Indianapolis, IN USA; 3 Department of Emergency Medicine, Wayne State University School of Medicine, Integrative Biosciences Center, Detroit, MI USA

**Keywords:** Sepsis, Echocardiography, SOFA score, Mortality, Emergency department, Mixed-effects models, Septic cardiomyopathy

## Abstract

**Background:**

Septic cardiomyopathy was recognized more than 30 years ago, but the early phase remains uncharacterized as no existing studies captured patients at the time of Emergency Department (ED) presentation, prior to resuscitation. Therapeutic interventions alter cardiac function, thereby distorting the relationship with disease severity and outcomes. The goal of this study was to assess the impact of illness severity on cardiac function during the first 24 h of sepsis admission.

**Methods:**

This was a pre-planned secondary analysis of a prospective observational study of adults presenting to the ED with suspected sepsis (treatment for infection plus either lactate > 2 mmol/liter or systolic blood pressure < 90 mm/Hg) who received < 1L IV fluid before enrollment. Patients had 3 echocardiograms performed (presentation, 3, and 24 h). The primary outcome was the effect of increasing sepsis illness severity, defined by ED Sequential Organ Failure Assessment (SOFA) score, on parameters of cardiac function, assessed using linear mixed-effects models. The secondary goal was to determine whether cardiac function differed between survivors and non-survivors, also using mixed-effects models.

**Results:**

We enrolled 73 patients with a mean age of 60 (SD 16.1) years and in-hospital mortality of 23%. For the primary analysis, we found that increasing ED SOFA score was associated with worse cardiac function over the first 24 h across all assessed parameters of left-ventricular systolic and diastolic function as well as right-ventricular systolic function. While baseline strain and E/e' were better in survivors, in the mixed models analysis, the trajectory of Global Longitudinal Strain and septal E/e′ over the first 24 h of illness differed between survivors and non-survivors, with improved function at 24 h in non-survivors.

**Conclusions:**

In the first study to capture patients prior to the initiation of resuscitation, we found a direct relationship between sepsis severity and global myocardial dysfunction. Future studies are needed to confirm these results, to identify myocardial depressants, and to investigate the link with adverse outcomes so that therapeutic interventions can be developed.

**Supplementary Information:**

The online version contains supplementary material available at 10.1186/s13089-022-00282-6.

## Introduction

Septic cardiomyopathy (SC) is a widely recognized yet ill-defined phenomena of myocardial dysfunction in patients with sepsis. SC has become an increasingly investigated constellation of pathologies, although it lacks formal diagnostic criteria [1, 2]. The importance of variability in myocardial performance in those dying compared to those surviving sepsis has long been recognized [3, 4]. A variety of imaging and biomarker approaches have been investigated, but results thus far are conflicting and ultimately inconclusive, leaving SC as a poorly defined entity [5–10]. While mortality due to sepsis has improved dramatically since SC was first recognized, further description of the pathophysiologic effects of sepsis on the heart is needed, with the ultimate goal of further improvement in patient outcomes.

Transthoracic echocardiography has become the preferred imaging modality for the study of SC given its ubiquity, ease and rapidity of use, and ability to reassess cardiac function over time. However, myocardial function has not been evaluated for the purposes of characterizing SC beginning at the time of presentation to the Emergency Department (ED). Instead, the current body of literature has been generated during Intensive Care Unit (ICU) stay and has not yet included cardiac function at the time of initial hospital arrival. This represents a substantial limitation as baseline function and the effects of early treatment are missed. Administration of large volumes of intravenous fluid (IVF) and vasoactive medications—cornerstones of sepsis management [11–13]—can profoundly alter cardiac function.

A crucial first step to address and manage SC involves a thorough functional description, beginning at the time of ED presentation. In fact, identification of unique sepsis phenotypes and the associated treatment responses, clinical trajectories, and outcomes has been identified by the National Institute of General Medical Sciences (NIGMS) as a priority for sepsis research [14]. Therefore, the goal of this study is to assess the relationship between cardiac function in the first 24 h of admission for sepsis and severity of illness as measured by Sequential Organ Failure Assessment (SOFA) score. Exploratory aims include assessment of the relationship between cardiac function and in-hospital mortality.

## Materials and methods

We performed a secondary, pre-planned, exploratory analysis from a prospective observational cohort study. The goal of the original study was to assess the association between diastolic function at the time of Emergency Department (ED) presentation and adverse outcomes in patients presenting with suspected sepsis. The study was performed at an urban Level 2 trauma center with an annual census of 100,000 visits. Inclusion criteria were suspected infection in patients ≥ 18 years of age plus either lactate > 2 mmol/liter or systolic blood pressure ≤ 90 mm/Hg. Patients were excluded if they had received > 1 L of IVF prior to enrollment, were pregnant, or incarcerated. A final diagnosis of sepsis was confirmed by manual chart review after discharge date by two independent investigators; in cases of disagreement, a third investigator reviewed the patient record and helped adjudicate the dispute. Patients were enrolled as a convenience sample when a qualified sonographer was available (a registered diagnostic cardiac sonographer or a study investigator with Emergency Ultrasound fellowship training). Informed consent was obtained from patients or a legally authorized representative; the study was approved by the institutional review board (M1 committee, protocol #1602014673). This manuscript was prepared in accordance with the Strengthening the Reporting of Observational Studies in Epidemiology (STROBE) guidelines (Additional file 1) [15].

A GE Vivid q ultrasound system (GE Healthcare, Milwaukee, WI) with a phased-array transducer and ECG-gating was used for image acquisition and images were stored for later offline interpretation on an EchoPAC workstation. The treating clinicians were blinded to study echocardiogram results, but could perform their own ultrasound examinations (including echocardiography) at their discretion. Patients had a total of 3 point-of-care echocardiograms performed: the first was done as soon as possible after arrival (T0), the next done 3 h after the first (T3), and the final examination done 24 h after the first (T24). Echocardiograms were interpreted by two study investigators who are both testamurs of the Examination of Special Competence in Adult Echocardiography administered by the National Board of Echocardiography and have extensive experience in point-of-care echocardiography research. They were blinded to clinical data and outcome status at the time of image interpretation. Images were acquired from the sub-costal, parasternal long, parasternal short, and apical (4-chamber, 2-chamber, and long-axis) windows. Left-ventricular ejection fraction (LVEF) was assessed by visual estimation after review of all images in increments of 5%. Parameters of diastolic function assessed included trans-mitral inflow velocity (E) with pulsed-wave Doppler, and septal and lateral mitral annular velocity (e′) using tissue Doppler [16]; sample volume location for diastolic assessment was performed in accordance with American Society for Echocardiography (ASE) guidelines [17]. In patients with atrial dysrhythmias, diastolic function parameters were assessed by averaging measures over 3 beats; E velocity was not measured when there was E-A fusion and these images were excluded from analysis. Tricuspid annular plane systolic excursion (TAPSE) was assessed using M-mode with the cursor placed at the lateral tricuspid annulus. Any images that were felt to be technically inadequate for the above measurements by either interpreter were excluded from analysis.

Speckle-tracking echocardiography was used to measure global longitudinal strain (GLS) from the 3 apical windows using the Automated Function Imaging (AFI) tool on the EchoPAC workstation. Manual repositioning of the AFI-derived endocardial border tracing was only performed when noted by interpreting physicians to be grossly malpositioned. Images were excluded from GLS analysis when the frame rate was outside the 40–80 frames-per-second range, heart rate was > 120 beats-per-minute, atrial dysrhythmias were present, aortic valve closure time could not be ascertained from the ECG tracing, or > 1 segment was non-trackable. GLS was calculated as the mean strain from each tracked myocardial segment. Diameter and collapsibility of the inferior vena cava (IVC) was measured from the sub-costal view in accordance with ASE guidelines [18]. Images captured more than 1 h after the time goal at T3 and T24 were also excluded to avoid potential confounding by additional time for therapeutic interventions and/or disease progression.

A REDCap database was used to store all study data, with abstraction from the electronic health record performed by trained research personnel, in accordance with prior guidelines [19]. Clinical data points collected included patient demographics, vital signs, past medical history, in-hospital mortality, ICU admission, use of mechanical ventilation, and hospital length-of-stay (LOS). Severity of illness was assessed using the SOFA score at ED presentation and again at 24 h (using worst values in that time period after ED measurement). Parameters that were not measured initially were entered as normal (0); for non-ventilated patients, FiO_2_ was calculated by previously described methods [20].

### Statistical analysis

Descriptive data are reported as means with standard deviation (SD), medians with inter-quartile range (IQR) or proportions with 95% confidence intervals (CI). The primary objective of the study was to determine the impact of illness severity, as measured by ED SOFA score, on cardiac function during the first 24 h of admission. To test the hypothesis that more severe disease is associated with greater degree of cardiac dysfunction, the relationship between echocardiography variables and clinical variables was explored using linear mixed-effects models. Repeated measures of each echocardiography variable (T0, T3, T24) were used as the dependent variable, with one model for each echocardiography parameter. The primary predictor of interest was ED SOFA score. A random intercept for individual patients was included in all models. Additional covariates were selected “by meaning” and included age, gender, ED troponin-I value, history of heart failure (either reduced or preserved systolic function), and volume of IVF administered during the first 24 h. In addition, baseline IVC collapsibility (≤ 50% versus > 50%) was included as a covariate as a proxy for preload. Lactic acid was not included as a covariate as SOFA score is felt to be a more detailed marker of illness severity. The exploratory goal was to determine if cardiac function differs between patients who die in-hospital compared to those who survive. To test this hypothesis, in-hospital mortality was added as a covariate to the aforementioned models. An interaction between time and in-hospital mortality was also included to assess whether the trajectory of cardiac function differs for survivors compared to non-survivors. When the interaction terms were statistically significant, interaction plots were created with model-predicted echocardiography variables on the y-axis, plotted against time on the x-axis, with a separate curve for survivors and non-survivors. To allow for a non-linear trajectory for each echocardiography parameter in the mortality models, time was modeled using a restricted cubic spline with 4 knots, at quantiles 0.05, 0.35, 0.65, and 0.95 [21]. Model fit and performance were ascertained by visual inspection of residual plots and using Akaike’s Information Criteria (AIC). Covariance structure for random effects was selected using AIC and the null model likelihood ratio test. Statistical analysis was performed using SAS (PROC MIXED) version 9.4 (SAS Institute, Cary, NC) and RStudio (packages “lme4” and “gtsummary”) version 1.3.1093 (RStudio Team, Boston, MA) with two-tailed significance set at 0.05. Given that this was a secondary analysis of previously collected data, no sample size calculation was performed.

## Results

A total of 73 patients were enrolled between August, 2018 and March, 2020. All patients were African-American. The overall cohort included 28 females (38%) with a mean age of 60.7 (SD 15.6 years); 14 patients (19%) had a history of HF. In-hospital mortality was 23% (*n* = 17), with 29 patients (39%) admitted to the ICU, 19 (26%) requiring vasopressors at any time during the first 24 h of admission, and 19 (26%) requiring mechanical ventilation. Median hospital LOS was 6.5 days (IQR 7). In terms of IVF administration, the median volume at 3 h was 2.0 L (IQR 2.0 L), and at 24 h was 4.5 L (IQR 3.0L) at 24 h.

Baseline patient characteristics, overall and stratified by in-hospital morality status, are listed in Table 1. Vital signs were similar between groups at presentation, while non-survivors were slightly older, male, and had greater co-morbidity burden. In non-survivors, SOFA scores were slightly greater in the ED, but substantially greater at 24 h, compared to the survivor group. Resuscitative IVF volumes at 3 and 24 h were similar, as was the proportion of patients receiving 30 cc/kg by 3 h. For ED SOFA data, 22 patients (*n* = 6 for non-survivors) did not have bilirubin and 44 (*n* = 5 for non-survivors) did not have PaO_2_ obtained and thus were scored as “0” for these categories. For the cardiovascular components of the SOFA score, in the ED 32 patients had a mean arterial pressure (MAP) < 70 mm/Hg (1 point), 6 had norepinephrine infusion at ≤ 1 mcg/kg/h (3 points), and 6 had norepinephrine infusion at > 1mcg/kg/h (4 points). At 24 h, 18 patients had MAP < 70 mm/Hg, 5 had norepinephrine infusion at ≤ 1 mcg/kg/h, and 7 had norepinephrine infusion at > 1mcg/kg/h (note that patients meeting criteria at each time point are not necessarily the same individuals). Only 7 patients (*n* = 1 for non-survivors) had NT-pro BNP measured.

**Table 1:** Baseline patient characteristics

	In-hospital mortality		Overall cohort
	Yes (*N* = 17)	No (*N* = 56)	*N* = 73
Age*	66.3 (12.9)	60.2 (14.7)	60.7 (15.6)
Female	4 (23, 1–46)	24 (43, 31–57)	28 (38, 28–51)
ED systolic BP	102 (33)	103.5 (39)	103 (37)
ED diastolic BP	66 (24)	68.0 (27)	64.7(27)
ED heart rate*	104 (21)	103 (24)	103 (23)
ED respiratory rate	20 (4)	20 (6)	20 (6)
ED Sp0_2_	98 (3)	98 (4)	98 (3)
ED temperature	36.9 (2)	36.9 (1.2)	36.9 (1.5)
Weight (kg)*	70.6 (12.3)	74.5 (17.1)	74.9 (17.3)
Past medical history			
Diabetes	10 (59, 36–78)	21 (38, 26–51)	31 (42, 32–54)
Stroke	5 (29, 13–53)	11 (20, 11–32)	16 (22, 14–33)
End-stage renal disease	6 (35, 17–59)	13 (23, 14–36)	19 (26, 17–37)
Coronary artery disease	4 (24, 10–47)	14 (25, 15–38)	18 (25, 16–36)
Cancer	9 (53, 31–73)	17 (30, 19–43)	26 (36, 26–47)
COPD/asthma	4 (24, 10–47))	12 (21, 12–34)	16 (22, 14–33)
Heart failure (pEF or rEF)	5 (29, 13–53)	9 (16, 9–28)	14 (19, 12–30)
Laboratory results and interventions			
ED Lactate (mmol/L)	4.3 (2.9)	2.9 (1.9)	3.1 (2)
ED Troponin-I (ng/mL)	0.07 (0.07)	0.05 (0.05)	0.06 (0.05)
NT-pro BNP (pg/mL)	1562 (0)	135.5 (107)	180 (555)
Hemoglobin (mg/dL)	10.4 (2.55)	11.2 (4.1)	11 (3.3)
Creatinine (mg/dL)	2.69 (2.77)	2.05 (3.04)	2.06 (2.76)
IVF 3 h (L)	2.0 (1.5)	2.0 (2.0)	2.0 (2.0)
IVF 24 h (ml)	3600 (2800)	3145 (3100)	4.5 (3.0)
30 cc/kg in 1st 3 h	10 (59, 30–90)	28 (58, 37–65)	38 (52,
SOFA score: ED	5 (4)	3 (5)	3 (5)
SOFA score: 24 h	6.5 (7)	2 (5)	3 (5)
Mechanical ventilation	9 (53, 31–74)	10 (18, 10–30)	19 (26, 17–37)
Vasopressor use	9 (53, 31–74)	10 (18, 10–30)	19 (26, 17–37)
Days in Hospital	6 (10)	7 (7)	6.5 (7.0)
Primary source of infection# (%, N)			
Pneumonia	41 (7)	34 (19)	36 (26)
GI/GU	35 (6)	38 (21)	37 (27)
Skin/soft tissue/bone	6 (1)	3 (2)	4 (3)
Other	18 (3)	25 (14)	23 (17)

Proportions are given as N (%, 95% CI); continuous variables given as median (inter-quartile range) except as denoted by *, which are given as mean (standard deviation)

*BP*  blood pressure; *COPD*  chronic obstructive pulmonary disease; *LVEF*  left-ventricular ejection fraction; *pEF/rEF*  preserved/reduced LVEF; *TAPASE*  tricuspid annular plan systolic excursion; *Avg e′*  average of septal and lateral mitral annular velocities; *IVF*  intravenous fluid; *SOFA*  sequential organ failure assessment; *ED*  emergency department; *GI*  gastrointestinal; *GU*  genitourinary; *Other*  indwelling vascular access device, bacteremia, or source unknown

^#^Some patients had more than one infectious source identified on chart review—only the primary source is listed here

A summary of echocardiographic variables at each timepoint (treating each examination as an independent, non-repeated event) is listed in Table 2. LV systolic function was slightly worse for non-survivors, with a greater magnitude of difference for GLS than for LVEF. Mitral annular velocities were similar between groups across all time points. For septal and average E/e′, non-survivors demonstrated improvement from 0 to 24 h whereas survivors had relatively stable measures; this trend was not seen for lateral E/e′. TAPSE and IVC measures were similar between groups.

**Table 2:** Echocardiographic parameters at each time point

Echocardiographic parameter			
	Dead (*n* = 17)	Alive (*n* = 56)	Overall (*n* = 73)
LVEF (%): hour 0	45 (22, 57)	55 (50, 65)	55 (45, 60)
LVEF (%): hour 3	48 (22, 60)	55 (49, 61)	55 (45, 60)
LVEF (%): hour 24	45 (40, 65)	50 (45, 60)	50 (45, 60)
GLS (%): hour 0	− 8.80 (− 11.90, − 5.70)	− 13.00 (− 15.90, − 12.60)	− 13.10 (− 16.02, − 12.55)
GLS (%): hour 3	− 10.2 (− 8.9, − 11.9)	− 13.7 (− 17.9, − 9.5)	− 13.6 (− 12.0, − 15.5)
GLS (%): hour 24	− 11.60 (− 14.75, − 7.20)	− 14.35 (− 14.80, − 13.12)	− 14.0 (− 15.1, − 12.0)
TAPSE (cm): hour 0	1.80 (1.80, 2.30)	2.00 (1.56, 2.45)	2.00 (1.72, 2.38)
TAPSE (cm): hour 3	2.20 (1.99, 2.46)	2.00 (1.67, 2.32)	2.10 (1.75, 2.50)
TAPSE (cm): hour 24	2.00 (1.20, 2.40)	2.00 (1.65, 2.32)	2.00 (1.50, 2.38)
IVC (cm): hour 0	1.05 (0.83, 1.70)	1.20 (0.90, 1.78)	1.25 (0.90, 1.72)
IVC (cm): hour 3	1.10 (0.75, 1.85)	1.60 (1.20, 2.10)	1.40 (1.05, 2.05)
IVC (cm): hour 24	0.95 (0.48, 1.87)	1.60 (1.25, 1.85)	1.60 (1.20, 1.90)
Lateral e′ (m/s): hour 0	0.06 (0.06, 0.08)	0.08 (0.07, 0.10)	0.08 (0.06, 0.10)
Lateral e′ (m/s): hour 3	0.07 (0.06, 0.09)	0.08 (0.06, 0.11)	0.08 (0.06, 0.11)
Lateral e′ (m/s): hour 24	0.06 (0.05, 0.08)	0.08 (0.07, 0.10)	0.08 (0.06, 0.10)
Septal e′ (m/s): hour 0	0.05 (0.04, 0.06)	0.07 (0.06, 0.08)	0.07 (0.05, 0.08)
Septal e′ (m/s): hour 3	0.06 (0.04, 0.07)	0.08 (0.05, 0.09)	0.070 (0.05, 0.09)
Septal e′ (m/s): hour 24	0.06 (0.05, 0.07)	0.07 (0.05, 0.08)	0.07 (0.05, 0.08)
Average e′ (m/s): hour 0	0.06 (0.05, 0.07)	0.08 (0.06, 0.1)	0.07 (0.06, 0.09)
Average e′ (m/s): hour 3	0.06 (0.05, 0.09)	0.08 (0.06, 0.10)	0.08 (0.06, 0.10)
Average e′ (m/s): hour 24	0.06 (0.06, 0.06)	0.08 (0.06, 0.09)	0.08 (0.06, 0.09)
Lateral E/e′: hour 0	9.1 (7.9, 14.9)	8.2 (6.2, 11.8)	8.4 (6.4, 11.5)
Lateral E/e′: hour 3	10.9 (8.6, 12.8)	8.4 (5.7, 12.7)	8.8 (5.9, 12.7)
Lateral E/e′: hour 24	9.7 (5.8, 12.8)	8.4 (5.7, 10.8)	8.4 (5.9, 11.8)
Septal E/e′: hour 0	14 (9, 19)	10 (7, 13)	10 (8, 14)
Septal E/e′: hour 3	10.4 (9.0, 19.0)	9.9 (7.3, 13.2)	9.9 (7.3, 14.3)
Septal E/e′: hour 24	8.7 (7.3, 10.1)	9.8 (7.2, 16.3)	10.0 (7.3, 14.4)
Average E/e′: hour 0	11 (9, 14)	10 (7, 13)	9.5 (7.6, 12.7)
Average E/e′: hour 3	9.9 (8.3, 15.8)	9.8 (6.5, 13.4)	9.8 (6.8, 13.2)
Average E/e′: hour 24	7.1 (7.1, 11.5)	8.7 (6.8, 13.8)	8.9 (6.9, 13.5)

Data are presented as median value (Q1, Q3) at each timepoint, with each cell representing an independent measurement (i.e., no within-subject correlation)

*LVEF* left-ventricular ejection fraction; *GLS* global longitudinal strain; *TAPSE* tricuspid annular plane systolic excursion; *IVC* maximal diameter of the inferior vena cava

### Mixed-models analysis

After adjusting for age, biologic sex, history of heart failure, ED troponin-I, volume of IVF received in 24 h, and baseline IVC collapse, we found that increase in ED SOFA score was associated with declining cardiac function across all parameters measured. Table 3 shows the mean change in each echocardiographic measure for every 1-point increase in ED SOFA score. The effect of IVF volume on the marginal mean was not significant in any of the models examined (data not shown). The overall effect sizes were modest, particularly for mitral annular velocities. Examination of residual variance for each model revealed that in all but 2 models, the majority of variation in each parameter of cardiac function not explained by the fixed effects exists within—rather than between—patients. As seen in Table 3, the measures with the greatest percent of variation explained by within-subject factors were E/e′, LVEF, and GLS.

**Table 3:** Mixed-effects model results showing the relationship between emergency department Sequential Organ Failure Assessment Score and marginal mean of echocardiography parameters

	ED SOFA score			
	Parameter estimate	95% CI	Percent residual variation not explained by within-subject effects	Number of observations included (%)
Echocardiography measure				
LVEF (%)	− 1.84	− 2.88 to − 0.80	59.5	185 (84)
TAPSE (mm)	− 0.72	− 1.2 to − 0.50	39.6	103 (47)
GLS (%)	0.30	− 0.01 to 0.61	84.3	85 (39)
Lateral e′(cm)	− 0.04	− 0.1 to − 0.02	31.2	147 (67)
Septal e′ (cm)	− 0.04	− 0.05 to − 0.01	53.0	149 (68)
Average e′ (cm)	− 0.04	− 0.10 to − 0.01	52.2	139 (63)
Lateral E/e′	0.98	0.29 to 1.67	71.3	126 (58)
Septal E/e′	1.28	0.44 to 2.11	73.1	126 (58)
Average E/e′	1.13	0.37 to 1.88	75.0	126 (58)

Each row represents a single linear mixed-effects model with the outcome being repeated measures (at 0, 3 and 24 h) of the cardiac function parameters listed in column 1 and the parameter estimate for ED SOFA score (covariate parameter estimates not shown)

*LVEF* left-ventricular ejection fraction; *TAPSE* tricuspid annular plan systolic excursion; *GLS* global longitudinal strain; *e′* mitral annular velocity; *E/e* trans-mitral inflow velocity/mitral annular velocity ratio

### In-hospital mortality

**Fig. 1:**
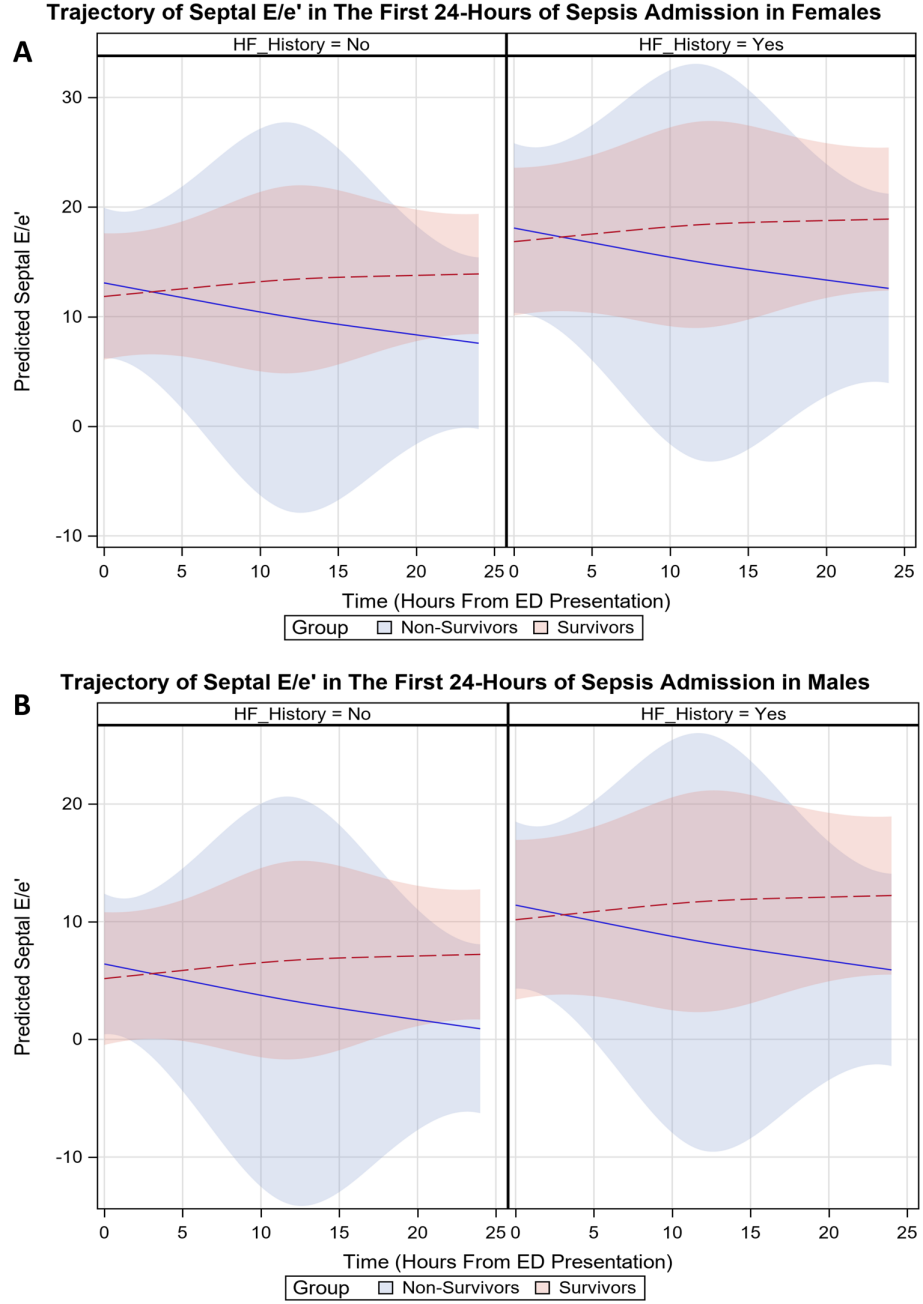
Trajectory of model-predicted septal E/e′ during the first 24 h of admission. Time–death interaction plot showing a different trajectory of predicted septal E/e′ in survivors versus non-survivors, adjusted for age, biologic sex, ED SOFA score, ED Troponin-I, IV volume given in 24 h, and baseline IVC collapsibility, stratified by heart failure history; bands represent 95% CIs. **A** depicts the relationship in females; **B** depicts the relationship in males

**Fig. 2:**
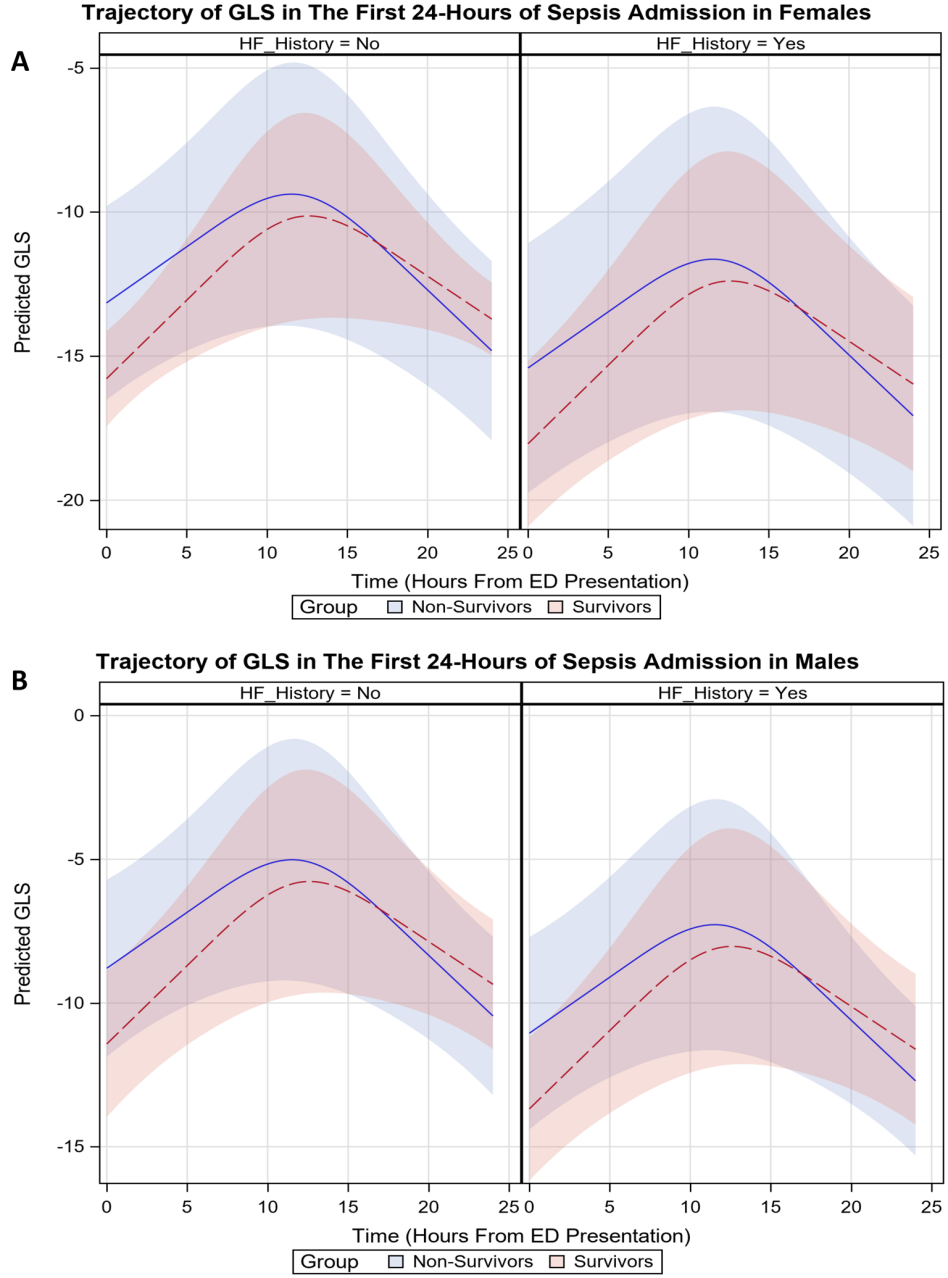
Trajectory of model-predicted global longitudinal strain during the first 24 h of admission. Time–death interaction plot showing a different trajectory of predicted GLS in survivors versus non-survivors, adjusted for age, biologic sex, ED SOFA score, ED Troponin-I, IV volume given in 24 h, and baseline IVC collapsibility, stratified by heart failure history; bands represent 95% CIs. **A** depicts the relationship in females; **B** depicts the relationship in males

Of the parameters investigated, only septal E/e′ and GLS were significantly different in survivors versus non-survivors, after adjustment for ED SOFA score, age, sex, ED troponin-I, IVF volume in 24 h, history of heart failure, and IVC collapsibility at presentation. There was a significant death–time interaction for both parameters (*p* = 0.03 for E/e′ and *p* = 0.02 for GLS). As shown in Fig. 1 (stratified by patients with versus without a history of HF), survivors show gradually increasing E/e′ while non-survivors show slight improvement. Females show overall greater E/e′ than males, and those with HF history show greater E/e′ than those without a history of HF. As seen in Fig. 2, GLS gradually worsens for both survivors and non-survivors until ~ 12 h before showing improvement towards 24 h, with survivors ending with slightly worse function compared to baseline whereas non-survivors showed slight improvement. Females and patients with a history of HF had better baseline GLS than males and those without history of HF.

## Discussion

In this first study to assess cardiac function at the time of presentation, prior to initiation of resuscitation, we found that increasing illness severity is independently associated with impaired cardiac function during the first 24 h of treatment for sepsis. While the magnitude of effect varied, the direct relationship was consistently seen for all measures of LV and RV systolic, as well as LV diastolic function. In terms of our secondary, exploratory outcome, we found that GLS and septal E/e′ were different in survivors versus non-survivors.

Cardiac dysfunction in sepsis has been recognized for decades [3, 4]. Existing literature is somewhat limited by the fact that prior studies have been performed in the ICU [1, 22, 23], thereby missing the early, pre-resuscitative phase of natural disease progression as well as the influence of initial therapeutic interventions. Earlier investigations have focused on identifying which parameters of cardiac function are most predictive of adverse outcomes, primarily death. In 1984, Parker et al. reported that in 20 patients with sepsis, LVEF was lower in survivors compared to non-survivors, but subsequent studies reported varied relationship between LVEF and survival [24, 25]. Similar findings have been reported across other echocardiographic parameters [1, 22, 23]. While the tacit belief has become that SC is characterized by impaired cardiac function and that said impairment portends a worse prognosis, neither of these beliefs have been objectively verified. A deleterious impact of IVF administration on cardiac function has also been posited to contribute to the development of SC [26, 27], but no significant contribution was seen in our study. Similarly, in a previously published study from this same cohort, we found that while patients with LVEF ≤ 40% were sicker than those with LVEF > 40%, both groups received similar IVF volumes and had similar outcomes [28]. Thus, our results represent an important contribution to the study of SC as the first longitudinal description of the disease, including assessment of cardiac function at the time of ED presentation, prior to initiation of any treatments.

Our findings support the idea that SC is a state of reduced cardiac function and further, that a dose–response relationship appears to exist between the severity of illness (SOFA score) and the degree of cardiac dysfunction. The latter results are consistent with the hypothesis that circulating myocardial depressants, released in response to infection, mediate the observed functional suppression. Numerous candidate molecules have been investigated (cytokines, reactive oxygen species, bioactive lipids) [22, 29, 30] but no clear causative agents have been identified. These biomarker studies, however, have the same limitations as echocardiography studies in that they have largely been performed in the ICU after substantial resuscitative interventions [5–10], including antibiotics, which are likely to change the inflammatory milieu and thus distort the relationship with cardiac function. In further support of this hypothesis, we found that after accounting for the influence of fixed effects (i.e., covariates) each echocardiographic parameter, the majority of the unexplained (i.e., residual) variance exists within individual patients. While not conclusive, these results again suggest the presence of a relatively uniform process contributing to myocardial dysfunction within each individual. Confirmatory studies, with concurrently obtained imaging and biomarker data, are needed to confirm these findings.

In terms of our exploratory outcome, we found that GLS varies over the first 24 h. While overall slightly worse in non-survivors at presentation, by 24 h GLS was improved in non-survivors but was worsened in survivors. Prior reports of GLS in sepsis have found that reduced GLS is common [31] and that it is a better predictor of adverse outcomes than LVEF [1, 32]. However, associations with mortality are mixed with some studies finding that impaired GLS is associated with increased mortality [33, 34] while others report no such association [10, 35, 36]. A notable difference of our study is the use of serial measures, begun at the time of presentation, that also accounts for IVF volume and other treatments, which may give a more complete description of the relationship between GLS and mortality. However, the influence of loading conditions on GLS, which are altered by treatments, necessitates cautions interpretation of these findings as observed changes are likely due to a combination of treatments administered as well as disease progression. While the interaction term was significant in the full model, the confidence limits on the interaction plots overlap, reflecting low precision from the small sample size.

For diastolic function, we also found a time-varying relationship between septal E/e′ and mortality, with survivors showing slightly worsening function over the first 24 h. Non-survivors had a slight improvement during this time, with the largest magnitude of change occurring in the first 12 h after presentation. More data are needed to better characterize the timing of change in cardiac function as our results represent estimated change based on 3 measurement points. However, the change appears to occur early in the treatment course thereby supporting our belief that data captured after hospital admission (as in prior studies) likely incompletely describe SC—both absolute level of function and trajectory are important.

The overall effect size of E/e′ was modest (the largest being ~ 4- to 5-unit change, with wide CIs), a relatively small difference which is of unclear clinical significance. However, the decline brought nearly all non-survivors below the reference standard of > 14 indicating elevated LV filling pressure [17], while survivors approached or exceeded this threshold. The hypothesis of a protective “hibernating myocardium” effect during times of physiologic stress [37–39] would support this finding. LV systolic function, as measured by GLS, was also worse at 24 h as compared to baseline, lending further support to this hypothesis. Due to our small, non-random sample, these results should be viewed as preliminary and the overall relationship between cardiac function and mortality requires further exploration.

In contrast to our results, several prior studies have reported that elevated E/e′ is associated with increased mortality [6, 9, 40], while one study found no association [41]. The former 3 studies also reported that reduced e′ velocity was associated with increased mortality when dichotomized to “normal” versus “abnormal” (variously defined as an e′ velocity from < 5 to < 8 cm/s). We found relatively small (and smaller than reported in prior investigations) between-group differences in these parameters (Table 2), likely leaving our study underpowered to detect a difference. Direct comparisons with our results are also somewhat difficult as we treated e′ as a continuous variable measured serially rather than at a single point after admission, and prior studies also did not always specify whether septal, lateral, or average values were used. When reported, however, differences in independent associations with adverse outcomes were seen in septal versus lateral measurements [6].

## Limitations

Our study has several limitations which must be considered when interpreting and applying results. Most notably, this is a small study done in a convenience sample of patients at a single institution. All of our participants were African-American, reflecting the demographics of our patient population. While a repeated-measures design does increase statistical power, replication of our findings in external populations is needed to confirm their veracity. Similarly, while we feel that our results and conclusions are strengthened by the longitudinal nature of our study design, it must be noted that the overall body of SC literature is quite varied in terms of echocardiographic findings and their relationship with outcomes. Clinical heterogeneity of patients, differences in when measurements occurred, what ultrasound equipment was used, and wide-ranging classifications of illness severity and degree of cardiac dysfunction all contribute to the widely varied findings. Although these issues are not unique to the study of SC—they plague many large-scale sepsis clinical trials [42]—future studies would benefit from collaborative efforts from researchers in this realm, with a focus on minimizing these potentially confounding influences. While the SOFA score is a validated overall measure of sepsis illness severity, points in each organ system may have varied effects on cardiac function. Administration of vasoactive medications may improve cardiac function even while increasing SOFA score; the effects on the heart of 4 points from liver disease may be different from 4 points for renal disease. Ultimately, untangling these complex relationships is difficult, but they should be carefully considered in planning future, non-observational trials. Echocardiography-specific limitations are that pre-septic cardiac function was unknown, and many measures of cardiac function are load-dependent, particularly GLS [43]. While we attempted to address these by adjusting for history of heart failure and baseline IVC collapsibility, these are imperfect proxies and thus residual confounding due to changes in loading conditions on the heart may exist. Owing to technical limitations, such as tachycardia, echocardiographic variables were not available at all data points, which could induce bias as elevated heart rate may be related to severity of sepsis. The repeated measures design may ameliorate these effects to some extent as the models account for within-subject variability between missing data points. We did not measure cardiac output or other indicators of volume responsiveness. Finally, we used visually estimated, rather than calculated, LVEF. We adopted this approach for pragmatic purposes as visual estimation is easily completed at the bedside in nearly all patients (i.e., fewer technical limitations than calculated methods). Our goal was to contrast this more readily available measure of LV systolic function with assessment of GLS, which, while it has been found to be more prognostically useful than LVEF in many diseases [44], is much more challenging to obtain. A tradeoff exists between fidelity and feasibility and while use of calculated LVEF may produce different results, data would likely be available from fewer patients.

## Conclusion

In this longitudinal, prospective observational study of patients presenting to the ED with suspected sepsis, we found that increasing illness severity, as measured by SOFA score, was associated with worse cardiac function across the first 24 h of illness. While baseline GLS and E/e′ were worse in non-survivors, in the mixed-effects models, these parameters improved from presentation to 24 h in non-survivors, while they worsened for survivors. These findings suggest that SC is overall characterized by global myocardial depression and that early, repeated measures of cardiac function offers prognostically useful data as cardiac function may change in the early phase of treatment. Additional studies are needed to identify the factors responsible for myocardial depression so that targeted therapeutics can be developed.

## Supplementary Information

**Additional file 1:** STROBE Statement—Checklist of items that should be included in reports of cohort studies.

## Data Availability

The datasets used and/or analyzed during the current study are available to qualified individuals from the corresponding author on reasonable request, in accordance with local IRB policies.

## Abbreviations

ED: Emergency department
IVF: Intravenous fluid
L: Liter
ECG: Electrocardiogram
ASE: American Society for Echocardiography
LVEF: Left-ventricular ejection fraction
TAPSE: Tricuspid annular plan systolic excursion
GLS: Global longitudinal strain
AFI: Automated function imaging
IVC: Inferior vena cava
ICU: Intensive care unit
SOFA Score: Sequential Organ Failure Assessment Score
SD: Standard deviation
IQR: Inter-quartile range
CI: 95% Confidence interval
AIC: Akaike’s Information Criteria
HF: Heart failure
LOS: Length-of-stay

## References

[1] Ehrman RR, Sullivan AN, Favot MJ, Sherwin RL, Reynolds CA, Abidov A (2018). Pathophysiology, echocardiographic evaluation, biomarker findings, and prognostic implications of septic cardiomyopathy: a review of the literature. Critical Care (London, England).

[2] Martin L, Derwall M, Al Zoubi S, Zechendorf E, Reuter DA, Thiemermann C (2019). The septic heart: current understanding of molecular mechanisms and clinical implications. Chest.

[3] Parker MM, Shelhamer JH, Bacharach SL, Green MV, Natanson C, Frederick TM (1984). Profound but reversible myocardial depression in patients with septic shock. Ann Intern Med.

[4] Weisel RD, Vito L, Dennis RC, Valeri CR, Hechtman HB (1977). Myocardial depression during sepsis. Am J Surg.

[5] Ikonomidis I, Nikolaou M, Dimopoulou I, Paraskevaidis I, Lekakis J, Mavrou I (2010). Association of left ventricular diastolic dysfunction with elevated NT-pro-BNP in general intensive care unit patients with preserved ejection fraction: a complementary role of tissue Doppler imaging parameters and NT-pro-BNP levels for adverse outcome. Shock (Augusta, Ga).

[6] Landesberg G, Gilon D, Meroz Y, Georgieva M, Levin PD, Goodman S (2012). Diastolic dysfunction and mortality in severe sepsis and septic shock. Eur Heart J.

[7] Landesberg G, Jaffe AS, Gilon D, Levin PD, Goodman S, Abu-Baih A (2014). Troponin elevation in severe sepsis and septic shock: the role of left ventricular diastolic dysfunction and right ventricular dilatation*. Crit Care Med.

[8] Pulido JN, Afessa B, Masaki M, Yuasa T, Gillespie S, Herasevich V (2012). Clinical spectrum, frequency, and significance of myocardial dysfunction in severe sepsis and septic shock. Mayo Clin Proc.

[9] Sturgess DJ, Marwick TH, Joyce C, Jenkins C, Jones M, Masci P (2010). Prediction of hospital outcome in septic shock: a prospective comparison of tissue Doppler and cardiac biomarkers. Critical care (London, England).

[10] Zaky A, Gill EA, Lin CP, Paul CP, Bendjelid K, Treggiari MM (2016). Characteristics of sepsis-induced cardiac dysfunction using speckle-tracking echocardiography: a feasibility study. Anaesth Intensive Care.

[11] Mouncey PR, Osborn TM, Power GS, Harrison DA, Sadique MZ, Grieve RD (2015). Trial of early, goal-directed resuscitation for septic shock. N Engl J Med.

[12] Peake SL, Delaney A, Bailey M, Bellomo R, Cameron PA, Cooper DJ (2014). Goal-directed resuscitation for patients with early septic shock. N Engl J Med.

[13] Yealy DM, Kellum JA, Huang DT, Barnato AE, Weissfeld LA, Pike F (2014). A randomized trial of protocol-based care for early septic shock. N Engl J Med.

[14] https://grants.nih.gov/grants/guide/notice-files/NOT-GM-19-054.html. Accessed 26 Jan 2022

[15] von Elm E, Altman DG, Egger M, Pocock SJ, Gøtzsche PC, Vandenbroucke JP (2007). Strengthening the reporting of observational studies in epidemiology (STROBE) statement: guidelines for reporting observational studies. BMJ.

[16] Lanspa MJ, Gutsche AR, Wilson EL, Olsen TD, Hirshberg EL, Knox DB (2016). Application of a simplified definition of diastolic function in severe sepsis and septic shock. Critical Care (London, England).

[17] Nagueh SF, Smiseth OA, Appleton CP, Byrd BF, Dokainish H, Edvardsen T (2016). Recommendations for the evaluation of left ventricular diastolic function by echocardiography: an update from the American Society of Echocardiography and the European Association of Cardiovascular Imaging. J Am Soc Echocardiogr.

[18] Lang RM, Badano LP, Mor-Avi V, Afilalo J, Armstrong A, Ernande L (2015). Recommendations for cardiac chamber quantification by echocardiography in adults: an update from the American Society of Echocardiography and the European Association of Cardiovascular Imaging. Eur Heart J Cardiovasc Imaging.

[19] Gilbert EH, Lowenstein SR, Koziol-McLain J, Barta DC, Steiner J (1996). Chart reviews in emergency medicine research: Where are the methods?. Ann Emerg Med.

[20] Lambden S, Laterre PF, Levy MM, Francois B (2019). The SOFA score-development, utility and challenges of accurate assessment in clinical trials. Critical Care (London, England).

[21] Harrell FE, Harrell FE (2015). General aspects of fitting regression models. Regression modeling strategies. Springer series in statistics.

[22] Martin L, Derwall M, Al Zoubi S, Zechendorf E, Reuter DA, Thiemermann C (2018). The septic heart: current understanding of molecular mechanisms and clinical implications. Chest.

[23] Beesley SJ, Weber G, Sarge T, Nikravan S, Grissom CK, Lanspa MJ (2018). Septic cardiomyopathy. Crit Care Med.

[24] Huang SJ, Nalos M, McLean AS (2013). Is early ventricular dysfunction or dilatation associated with lower mortality rate in adult severe sepsis and septic shock? A meta-analysis. Critical Care (London, England).

[25] Sevilla Berrios RA, O'Horo JC, Velagapudi V, Pulido JN (2014). Correlation of left ventricular systolic dysfunction determined by low ejection fraction and 30-day mortality in patients with severe sepsis and septic shock: a systematic review and meta-analysis. J Crit Care.

[26] Kakihana Y, Ito T, Nakahara M, Yamaguchi K, Yasuda T (2016). Sepsis-induced myocardial dysfunction: pathophysiology and management. J Intensive Care.

[27] Marik PE, Lemson J (2014). Fluid responsiveness: an evolution of our understanding. Br J Anaesth.

[28] Ehrman RR, Ottenhoff JD, Favot MJ, Harrison NE, Khait L, Welch RD (2022). Do septic patients with reduced left ventricular ejection fraction require a low-volume resuscitative strategy?. Am J Emerg Med.

[29] Cioccari L, Luethi N, Masoodi M (2020). Lipid mediators in critically Ill patients: a step towards precision medicine. Front Immunol.

[30] Dalli J, Colas RA, Quintana C, Barragan-Bradford D, Hurwitz S, Levy BD (2017). Human sepsis eicosanoid and proresolving lipid mediator temporal profiles: correlations with survival and clinical outcomes. Crit Care Med.

[31] Boissier F, Razazi K, Seemann A, Bedet A, Thille AW, de Prost N (2017). Left ventricular systolic dysfunction during septic shock: the role of loading conditions. Intensive Care Med.

[32] Kalam K, Otahal P, Marwick TH (2014). Prognostic implications of global LV dysfunction: a systematic review and meta-analysis of global longitudinal strain and ejection fraction. Heart (British Cardiac Society).

[33] Chang WT, Lee WH, Lee WT, Chen PS, Su YR, Liu PY (2015). Left ventricular global longitudinal strain is independently associated with mortality in septic shock patients. Intensive Care Med.

[34] Palmieri V, Innocenti F, Guzzo A, Guerrini E, Vignaroli D, Pini R (2015). Left ventricular systolic longitudinal function as predictor of outcome in patients with sepsis. Circ Cardiovasc Imaging.

[35] De Geer L, Engvall J, Oscarsson A (2015). Strain echocardiography in septic shock—a comparison with systolic and diastolic function parameters, cardiac biomarkers and outcome. Critical Care (London, England).

[36] Orde SR, Pulido JN, Masaki M, Gillespie S, Spoon JN, Kane GC (2014). Outcome prediction in sepsis: speckle tracking echocardiography based assessment of myocardial function. Critical Care (London, England).

[37] Budinger GR, Duranteau J, Chandel NS, Schumacker PT (1998). Hibernation during hypoxia in cardiomyocytes. Role of mitochondria as the O2 sensor. J Biol Chem.

[38] Levy RJ, Piel DA, Acton PD, Zhou R, Ferrari VA, Karp JS (2005). Evidence of myocardial hibernation in the septic heart. Crit Care Med.

[39] Siddiqui Y, Crouser ED, Raman SV (2013). Nonischemic myocardial changes detected by cardiac magnetic resonance in critical care patients with sepsis. Am J Respir Crit Care Med.

[40] Rolando G, Espinoza ED, Avid E, Welsh S, Pozo JD, Vazquez AR (2015). Prognostic value of ventricular diastolic dysfunction in patients with severe sepsis and septic shock. Rev Bras Ter Intensiva.

[41] Brown SM, Pittman JE, Hirshberg EL, Jones JP, Lanspa MJ, Kuttler KG (2012). Diastolic dysfunction and mortality in early severe sepsis and septic shock: a prospective, observational echocardiography study. Crit Ultrasound J.

[42] Vincent JL, Rello J, Marshall J, Silva E, Anzueto A, Martin CD (2009). International study of the prevalence and outcomes of infection in intensive care units. JAMA.

[43] Nafati C, Gardette M, Leone M, Reydellet L, Blasco V, Lannelongue A (2018). Use of speckle-tracking strain in preload-dependent patients, need for cautious interpretation!. Ann Intensive Care.

[44] Potter E, Marwick TH (2018). Assessment of left ventricular function by echocardiography: the case for routinely adding global longitudinal strain to ejection fraction. JACC Cardiovasc Imaging.

